# Soil-transmitted helminth infections and nutritional indices among Filipino schoolchildren

**DOI:** 10.1371/journal.pntd.0010008

**Published:** 2021-12-22

**Authors:** Mary Lorraine S. Mationg, Gail M. Williams, Veronica L. Tallo, Remigio M. Olveda, Eindra Aung, Portia Alday, Mark Donald Reñosa, Chona Mae Daga, Jhoys Landicho, Maria Paz Demonteverde, Eunice Diane Santos, Thea Andrea Bravo, Franziska Angly Bieri, Yuesheng Li, Archie C. A. Clements, Peter Steinmann, Kate Halton, Donald E. Stewart, Donald P. McManus, Darren J. Gray

**Affiliations:** 1 Research School of Population Heath, The Australian National University, Canberra, Australia; 2 Department of Epidemiology and Biostatistics, Research Institute for Tropical Medicine, Manila, Philippines; 3 School of Public Health, University of Queensland, Brisbane, Australia; 4 St Vincent’s Clinical School, University of New South Wales, Sydney, Australia; 5 Molecular Parasitology Laboratory, Infectious Diseases Division, QIMR Berghofer Medical Research Institute, Brisbane, Australia; 6 Hunan Institute of Parasitic Diseases, World Health Organization Collaborating Centre for Research and Control on Schistosomiasis in Lake Region, Yueyang, China; 7 Faculty of Health Sciences, Curtin University, Perth, Australia; 8 Swiss Tropical and Public Health Institute, Basel, Switzerland; 9 University of Basel, Basel, Switzerland; 10 School of Public Health and Social Work, Queensland University of Technology, Brisbane, Australia; 11 School of Medicine, Griffith University, Brisbane, Australia; Hitit University, Faculty of Medicine, TURKEY

## Abstract

**Background:**

Soil-transmitted helminth (STH) infections are still prevalent among schoolchildren in the Philippines. We evaluated the risk factors associated with STH and the relationship between STH and nutritional indices among schoolchildren aged 9–10 years in Laguna province, the Philippines.

**Methods:**

We used the baseline data from 40 schools enrolled in a randomised controlled trial of the Magic Glasses Philippines health education package. Data on demographic and socio-economic variables, and STH related knowledge, attitudes and practices, were obtained through a questionnaire. Stool samples were collected and assessed for STH egg presence using the Kato-Katz technique. Haemoglobin levels and height and weight of study participants were also determined. The generalized estimating equations approach was used to construct logistic regression models to assess STH-associated risk factors, and the association between any STH infection and anaemia, child stunting, wasting and being underweight. The trial is registered with the Australian New Zealand Clinical Trials Registry (ACTRN12616000508471).

**Findings:**

Among 1,689 schoolchildren, the prevalence of any STH was 23%. The prevalence of anaemia, stunting, being underweight and wasting was 13%, 20.2%, 19% and 9.5%, respectively. Age, socio-economic status, rural/urban classification of schools and knowledge of STH were significant risk factors for acquiring a STH infection. Moreover, infections with any STH were significantly associated with stunting (*P* = <0.001) and being underweight (*P =* <0.003), but not wasting (*P =* 0.375) or anaemia (*P* = 0.462) after controlling for confounding covariates.

**Conclusion:**

The study findings emphasise the need for sustainable deworming in tandem with other measures such as the provision of health education, improvements in sanitation and hygiene, and nutritional programs in order to control STH infections and improve morbidity outcomes in schoolchildren.

**Trial registration:**

Australian New Zealand Clinical Trials Registry (ACTRN12616000508471).

## Author summary

Soil-transmitted helminth (STH) infections continue to be high among schoolchildren in the Philippines despite the conduct of semi-annual nationwide school-based mass drug administration (MDA) program more than a decade ago. In this cross-sectional survey, we assess the prevalence of STH, the risk factors associated with infection, and the impact of STH on nutritional indices among schoolchildren in Laguna province, the Philippines. Our results show that STH prevalence (23%) remains above the World Health Organization (WHO) target of < 20% for morbidity control. The prevalences of stunting (20.2%), being underweight (19%) or wasting (9.5%) are high and are still regarded as public health problems according to WHO criteria, but low anaemia prevalence (13%) was observed. Age, socio-economic status, rural/urban classification of schools and knowledge of STH were significant risk factors for STH infections. Moreover, infections with STH were significantly associated with stunting and being underweight but not wasting or anaemia after controlling for key confounders. Sustainable deworming in tandem with other measures such as the provision of health education, improvements in sanitation and hygiene, and nutritional programs, are needed to control intestinal worm infections and improve morbidity outcomes in schoolchildren.

## Introduction

Soil-transmitted helminth (STH) infections are the most prevalent of the neglected tropical diseases (NTD). They infect more than one billion individuals world-wide [1]. In 2021, the World Health Organization (WHO) declared ambitious targets to reduce the burden of STH by eliminating them as a public health problem by 2030 [2]. Morbidity due to STH has been accounted to range from 1.97–3.3 million disability-adjusted life years (DALYs) [3,4]; school-aged children usually experience the highest burden [5]. Morbidities associated with STH in children include malnutrition, impaired growth, anaemia, and cognitive and educational deficits [6,7].

In the Philippines, STH are highly prevalent and major health problem, particularly in schoolchildren [8]. STH prevalence reported from several studies across the country range from 33.8–75.9% [9–14]. This is despite the implementation of a semi-annual nationwide school-based mass drug administration (MDA) program that was initiated more than a decade ago [15,16]. Notwithstanding the considerable efforts undertaken in the Philippines to control STH through regular school-based MDA, the persistence of STH requires careful assessment of the risk factors associated with infection and the morbidity burden.

Data from a recent (2018) Philippines National Nutrition Survey by the Food and Nutrition Research Institute of the Department of Science and Technology (FNRIDOST) indicated that stunting (24.5%) and being underweight (25%) remain highly prevalent among 6–10 year old schoolchildren, while the prevalence of wasting was 7.6% [17]. Furthermore, the prevalence of anaemia has also increased, affecting some 23.5% of six-year old children [17], translating into a moderate public health problem based on WHO criteria [18].

There are several reports from the Philippines on the factors associated with malnutrition in children [19–23] but relatively few have described the impact of STH [13,24–26]. Furthermore, previous studies correlating STH infections with anaemia are conflicting [27–30]. As the impact of STH on children’s health has not been extensively reported in the Philippines, studies assessing STH-associated morbidity in children are therefore needed to effectively monitor the success of STH control program in country. The present study was undertaken to determine STH prevalence, the risk factors associated with infection, and the impact of STH on nutritional indices among schoolchildren in Laguna province, the Philippines.

## Methods

### Ethics statement

Ethical clearance was obtained from the Research Institute for Tropical Medicine (RITM) Institutional Review Board (approval number 2013–16), the Philippines, QIMR Berghofer Medical Research Institute Human Ethics Committee (approval number: P1271), Australia, and the Australian National University Human Ethics Committee (approval number: 2014/356). Permission to undertake the study was obtained from the Philippines Department of Education (DepEd) prior to the start of the study. Written informed consent was obtained from parents and guardians of children. Written assent was also obtained from students who agreed to participate. Parasitological and anaemia results were communicated to all parents at the end of the survey, with a recommendation to seek treatment from local health centres.

### Study setting, design and participants

This study was conducted as part of the baseline assessment for a cluster randomised controlled trial (RCT) of “The Magic Glasses Philippines”. The trial aimed to determine the impact and generalizability of the school-based health education package for the prevention of STH in Laguna province [31,32]. Laguna is located on the Island of Luzon, in the Calabarzon Region of the Philippines, with 3.3 million population as of the last census conducted in 2020. It is also considered as the 3^rd^ populous and densest province in the Philippines [33]. Due to the high STH infections (averages ~33%), as confirmed by our pilot survey in 2015 [11], we have selected this area as the study site for the main RCT. The 40 schools included in the study were selected using a spatial sampling technique and according to the inclusion criteria of minimum three kilometres distance from each other. Further details of the design of the RCT, the study setting and procedures are available in the published study protocol [31]. The study baseline survey was carried out (June and July 2016) on schoolchildren aged 9–10 years (Grade 4) in 40 selected schools.

The sample size for present study was bound by the sample size calculation for the main RCT. This was designed to have 80% power to detect the intervention effect, using an intervention efficacy of 30% [18]. Calculations assumed an infection prevalence of 18% and a design effect of 1.5 to account for the cluster effect, and a predicted annual 10% loss-to-follow-up. The sample size calculation determined the requirement of 20 clusters for each study group (40 in total), corresponding to 1,520 study participants (assuming 38 participants per cluster).

### Study procedures

#### KAP questionnaire

All study participants completed a questionnaire to collect data on demographics, and knowledge, attitude and practices (KAP) about STH infection at trial baseline [31]. Full details of the questionnaires and KAP survey procedures are provided in the published study protocol [31]. In brief, the KAP questionnaire included multiple-choice and open-ended questions on demographics, health characteristics, medical history, and previous health education; knowledge about intestinal worms, how they are transmitted, and the symptoms and treatment of STH infection; the student’s attitude toward STH; self-reported hygiene practices in relation to hand washing, handling of food, using the toilet, and wearing of shoes. The questionnaire was developed in English, translated to Tagalog and back-translated into English to ensure accuracy. It was also pre-tested in two schools outside the RCT trial area.

The questionnaire was administered to the children by two research staff members. The instructions and questions in the questionnaire are read one-by-one to the children in front of the class by the first staff whereas the other moved around the room to check whether the children were able to follow the instructions and to ensure that each question was answered. The research staff had undergone a one-week training on data collection procedures including the administration of the KAP questionnaire before the start of the survey [31].

The data on the household characteristics associated to household water source and household assets were collected in the first trial follow-up KAP survey and we assumed that the status of the household characteristics did not change in the 5 months following the baseline survey.

#### Parasitological examination

Schoolchildren were asked to provide two stool samples over a period of four collection days to the research staff at the school [31]. From each sample, triplicate Kato-Katz (KK) thick smears (41.7 mg of stool/smear) were prepared at the school site within two hours after collection and these were all examined the same day [34]. Eggs were counted for all STH species to record prevalence and infection intensity (eggs per gram of faeces; EPG). Intensity of infection for each species was interpreted as light, moderate or heavy based on WHO criteria [35]. Prevalence of STH was defined as the presence of at least one STH species or genus (i.e., *A*. *lumbricoides* and/or *T*. *trichiura* and/or hookworm). For quality control, 10% of all slides were randomly selected and re-examined by a reference microscopist on each collection day.

#### Nutritional status assessment

All participating children underwent a single measurement at baseline of height (to nearest 0.1 cm) using a height scale chart (paper beam chart) and weight (to nearest 0.1 kg) using a calibrated digital weighing scale (Tanita HD-383, Tanita Corporation, Japan). Indicators for malnutrition included height-for-age Z-score (HAZ) to assess stunting, body mass index (BMI)-for-age Z-score (BAZ) to assess wasting, and weight-for-age Z-score (WAZ) (children aged <10 years only) to assess underweight. The calculations were done using the SAS macros for 2007 WHO Child Growth Standards for school-aged children and adolescents [36]. Each of these continuous outcomes was categorised, with individuals classified as stunted, underweight or wasted if HAZ, WAZ and BAZ, respectively, were more than two standard deviations below the WHO growth reference values [37].

#### Haemoglobin concentration assessment

Finger-prick blood samples were collected from participating children using a portable haemoglobin analyser (HemoCue Hb 301 System, HemoCue Sweden). The haemoglobin level was classified according to the WHO definition of anaemia severity (i.e., non-anaemic, mild, moderate and severe) [38]. However, due to the skewed distribution of those categorized as having mild, moderate or severe anaemia, anaemia status was re-categorized as a binary variable (defined as anaemic/non-anaemic using the cut-off set by WHO [38]) and used as a primary outcome.

### Data analysis

Analyses to determine the association between STH infections and nutritional indices (i.e., stunting, wasting, underweight and anaemia) were undertaken using the baseline cross-sectional data from the 40 schools enrolled in the Magic Glasses Philippines RCT. The analyses included study participants with complete data on KAP, at least one stool sample, blood sample, height and weight at baseline, and with a first follow-up KAP at 6 months.

To establish the socio-economic status (SES) of the study participants, we used a household-based asset approach [39]. Employing principal component analysis (PCA), a household wealth index variable was constructed from nine indicators: cement or tile floor material, access to electricity, having a radio, television set, refrigerator, cell phone, bicycle, motorcycle or jeep, or a truck. Children were asked about the presence of each indicator in the household. The factor scores from the first principal component were used as a measure of SES [39]. The first principal component explained 21.7% of the total variability. Variables such as the type of toilet used and the source of available drinking water were excluded from the PCA analysis as they were considered risk factors for STH infection in their own right.

For the KAP questionnaire, each correct answer for the knowledge and attitude components carried a score of one mark, an incorrect answer received a minus one mark and a “don’t know” answer was scored zero. In the case of the practices component, “Never” was scored zero, while “Some of the time”, “Most of the time” and “Always” were scored as “1”, “2” and “3”, respectively. “Always” shows maximum frequency for an event, and therefore was assigned the highest score. The overall scores calculated for the knowledge, attitude and practice components in the KAP questionnaire were 31, 7 and 29, respectively. The respondents’ overall knowledge level and practice securing a score of 20 or more were categorized as having good, 11–19 scores as an average, and ≤10 scores as poor. For overall attitude score, respondents with a score of 6 or more were categorized as having a good attitude, 4–5 scores as an average attitude, and ≤3 scores as a poor attitude. Meanwhile, for the knowledge sub-component scores (i.e., overall knowledge score on STH transmission and signs and symptoms), respondents with a score of 5–6 were categorized as having good, 3–4 scores as an average, and ≤2 scores as a poor. For overall knowledge score on STH prevention, respondents with a score of 7–9 were categorized as having good, 4–6 scores as an average, and ≤3 scores as poor.

A generalized estimating equations (GEE) approach was used to construct logistic regression models to account for possible clustering at the school level. Logistic regression models were constructed to assess the following: 1) predictors of STH infections; 2) the association between stunting, being underweight and wasting odds, and STH infection status adjusting for age, SES, anaemia and rural/urban classification of schools (according to the Philippines Statistical Authority (PSA) rural/urban classification of villages where the schools were located); and 3) the association between anaemia and STH infection status adjusting for age, sex, SES and rural/urban classification of each school. For the model predicting the risk factors for STH infections, univariable regression was undertaken for each of the risk factors, with inclusion in the multivariable model if they had *P*<0.2 and then removed iteratively until only the variables significant at *P*<0.05 remained in the final model. For model assessing the associations between STH infection and nutritional indices; and between STH infection and anaemia, all potential confounders regardless of their significance in the univariate analysis were forced in the final model. For models of stunting, being underweight and wasting, anaemia was added because of its importance as a potential confounder.

All data management and analyses used SAS Proprietary Software 9.4 (TS1M3) [Copyright 2002–2012 by SAS Institute Inc., Cary, NC, USA, Licensed to AUSTRALIAN NATIONAL UNIVERSITY–EAS, Site 10004431].

## Results

### General characteristics of the study population

At baseline, 3,832 students from the 40 targeted schools consented to participate in the study. Of those providing consent, 1,774 had complete data on baseline KAP, at least one stool sample, finger-prick blood sample, and height and weight measurements. Of these 1,774 students, 1,689 (95%) had matching KAP data at first follow up and were included in the analysis.

More than half of the participating schoolchildren were female (54.1%); the majority (89.2%) were aged 7–9 years (median age of study participants at enrolment was 9 years; interquartile range [IQR], 8–9 years). The majority (73.2%) of the students attended urban schools. Almost half (46.5%) of the respondents reported sourcing their drinking water from a piped water source and 71.9% reported using a pour-flush or water-sealed toilet (Table 1).

**Table 1:** Demographic and household characteristics of schoolchildren in Laguna Province, Philippines (n = 1,689).

Characteristic	Number	%
**Age group**		
7–9 years old	1507	89.2
≥10 years	182	10.8
**Sex**		
Male	776	45.9
Female	913	54.1
**Household assets**		
Cement floor or tile (n = 1576)	1298	82.4
Access to electricity (n = 1612)	1572	97.5
Owning radio (n = 1585)	1140	71.9
Owning TV (n = 1610)	1542	95.8
Owning refrigerator (n = 1571)	974	62.0
Owning a cell phone (n = 1602)	1562	97,5
Owning bicycle (n = 1588)	1020	60.4
Owning motorcycle or jeep (n = 1576)	915	54.2
Owning truck (n = 1540)	385	25.0
**School urban/rural classification**		
Urban	1237	73.2
Rural	452	26.8
**Main source of water for drinking**		
Piped water	785	46.5
Well	40	2.4
Hand pump	151	8.9
Spring	90	5.3
River/pond	10	0.6
Other source (bottle water or mineral water)	524	31.0
No answer	89	5.3
**Type of toilet available**		
Flush	392	23.2
Pour-flush or water-sealed toilet	1215	71.9
Closed Pit toilet (Antipolo)	40	2.4
No answer	42	2.5

### Prevalence of STH and nutritional characteristics

Of the 1,689 schoolchildren included, 389 (23%) were infected with at least one STH species, with the prevalence for *A*. *lumbricoides*, *T*. *trichiura* and hookworm being 15.9%, 13.3%, and 0.12% respectively. Among these, 63.2%, 94.2% and 100% of the infected children had light intensity burdens of *A*. *lumbricoides*, *T*. *trichiura* and hookworm, respectively. Prevalences of stunting, being underweight or wasting were 20.2%, 19.0% and 9.5% respectively. Only 13.0% of the study population suffered from anaemia, with the majority of these (11.1%) being mildly anaemic, and 1.9% were severely anaemic (Table 2).

**Table 2:** STH prevalence and nutritional characteristics of schoolchildren in Laguna Province, the Philippines (n = 1,689).

Characteristic	Number	%
**Infection Status**		
Any STH prevalence	389	23.0
*Ascaris* prevalence	269	15.9
Light infection ^†^	170	63.2
Moderate to Heavy infection ^†^	99	36.8
*Trichuris* prevalence	224	13.3
Light infection^╪^	211	94.2
Moderate to Heavy infection^╪^	13	5.8
Hookworm prevalence	2	0.12
Light infection^╪╪^	2	100
**Haemoglobin level**		
Mean haemoglobin (g/L, (SD))	125.7 (11.8)	
Non-anaemic	1469	87.0
Mildly anaemic	188	11.1
Moderately/severely anaemic	32	1.9
**Height-for-age (HAZ) / stunting**		
Mean (SD)	-1.09 (1.1)	
Normal	1347	79.8
Stunted	342	20.2
**Weight-for-age (WAZ)/underweight (n = 1,520)** *		
Mean (SD)	-0.78 (1.4)	
Normal	1231	81.0
Underweight	289	19.0
**BMI-for-age (BAZ) / wasting**		
Mean (SD)	-0.34 (1.4)	
Normal	1529	90.5
Wasted	160	9.5

*For WAZ (an indicator of underweight), only calculated for individual ≤ 10 years of age (age group 10 years covers up to age 120 completed months), n = 1,520.

† Prevalence of light and moderate intensity among those infected with *Ascaris lumbricoides*

^╪^ Prevalence of light and moderate intensity among those infected with *Trichuris trichiura*

^╪╪^ Prevalence of light and moderate intensity among those infected with *hookworm*

Anaemia classification was according to WHO guidelines [38]. For children 5–11 years of age: normal (≥115 g/L), mild (110–114 g/L), moderate (80–109 g/L), severe (< 80/L); children 12–14 years of age: normal (≥120 g/L); mild (110–119 g/L), moderate (80–109 g/L), severe (<80 g/L); non-pregnant women (≥15 years of age): normal (≥120 g/L), mild (110–119 g/L), moderate (80–109 g/L), severe (<80g/L); men (≥15 years of age): normal (≥130 g/L), mild (110–129 g/L), moderate (80–109 g/L) and severe (<89 g/L).

### Knowledge of STH

Sixty-four percent of the schoolchildren in the study acknowledged having previously heard of intestinal worms; *A*. *lumbricoides* or roundworms was heard by 53.2% of the schoolchildren, while 44.5% and 15.6% knew about *T*. *trichiura* or whipworms and hookworm, respectively. The majority indicated nurse/doctor (53.0%), television (49.5%), parents (44.9%) or school (41.4%) as the source of this information. About 67.1%, 47.4% and 43.8% of children were categorized as having poor knowledge of STH signs and symptoms, STH transmission and prevention, respectively (Table 3).

**Table 3:** Knowledge on STH transmission, signs and symptoms of infection and preventive measures.

Knowledge characteristic	Number	%
**STH transmission**		
Eat dirty food	1067	63.2
Walk barefoot	1055	62.5
Long and dirty fingernails	999	59.1
Dirty hands	977	57.8
Playing with soil	971	57.5
Playing in dirty places	848	50.2
Swimming in the canal/river	767	45.4
Flies landing on wounds	502	39.7
Mosquito bite	219	13.0
Fishing	101	6.0
**Overall STH transmission knowledge**		
Poor	801	47.4
Average	621	36.8
Good	267	15.8
**Knowledge about STH signs and symptoms**		
Belly ache	1240	73.4
Diarrhoea	1063	62.9
Poor appetite	749	44.3
Slow Growth	562	33.3
Fever	542	32.1
Can’t concentrate at school	400	23.7
Feeling tired	334	19.8
Overweight	255	15.1
High blood pressure	198	11.7
Blindness	79	4.7
**Overall knowledge about signs and symptoms**		
Poor	1133	67.1
Average	497	29.4
Good	77	4.6
**Knowledge about STH prevention**		
Washing fruit before eating	1187	70.3
Washing hands before eating	1178	69.7
Not playing in dirt	1140	67.5
Washing hands after toilet	1096	64.9
Keeping up personal hygiene	1038	61.5
Cover food	755	44.7
Doing enough exercises	655	38,8
Always wear shoes or sandals	455	26.9
Using the latrine	445	26.3
Sleeping under a mosquito net	427	25.3
Not playing in vegetable garden	380	22.5
Eating too much	200	11.8
**Overall knowledge about STH prevention**		
Poor	740	43.8
Average	769	45.5
Good	180	10.7
**Overall knowledge score**		
Poor	510	30.2
Average	988	58.5
Good	191	11.3

The great majority (93.3%) of participating schoolchildren believed that STH infections are treatable. When asked where they should receive treatment, a majority answered health centre (78.90%) or hospital (70.5%), while a small proportion answered school (7.0%), parent (7.2%) or traditional healer (6.0%). A majority (63.4%) considered that STH treatment prevented reinfection. Overall the STH knowledge score indicated that more than half (58.5%) of the schoolchildren had an average knowledge of intestinal worms (Table 3).

### Attitude and practices of schoolchildren towards STH infection

The KAP questionnaire further revealed that the majority (86%) of the schoolchildren believed that they had a chance of STH infection; of these, 36.8% considered they had a medium chance of acquiring the infection. The majority (76.7%) reported that they would worry if they became infected. However, 42.5% of participants did not consider that STH infection would prevent them from attending school. Furthermore, 85.6% reported they were able to wash their hands after toilet use at school. Overall, 46.2% of the schoolchildren had an average attitude score regarding STH infection (see S1 Table for additional information).

In relation to reported STH prevention practices, 81.9% indicated they always wore slippers outside their home, wore slippers inside the house (43.5%) and wore shoes at school (79.8%). Most of the children reported that they always defecated in their home latrine (86.1%) while a small proportion indicated they used a public/shared latrine (4.1%), the field (2.7%), and river or canal (1.7%). The majority (74%) indicated they always washed hands after toilet use; and 81.9% indicated they always used soap when washing. Similarly, the majority (87.8%) indicated they always washed their hands before eating with 80.2% always using soap. The majority also indicated the practice of washing fruits before eating (88.3%), and covering leftover food (81.8%) at all times. In general, 91.1% of the study participants were categorized as having good self-reported practices towards STH prevention (see S1 Table for additional information).

### Factors associated with STH infection

Multivariate logistic regression using GEE modelling showed that increasing age (age as a continuous variable) was significantly associated with STH infection (Adjusted Odds Ratio (AOR: 1.5; 95% Confidence Interval [CI]: 1.3─1.8; *P* = <0.001). The SES score was also found associated with an increased odds of STH infection (AOR: 1.2; 95% CI: 1.1–1.3; *P* = 0.001). However, unexpectedly, children located in rural schools (AOR: 0.7; 95% CI: 0.5–0.9; *P* = 0.016) were significantly less likely to have a STH infection compared with urban schools. Moreover, children with poor (AOR: 1.2; 95% CI: 1.0–1.6; *P* = 0.185) and average knowledge of STH (AOR: 1.2; 95% CI: 1.1–1.5; *P* = 0.013) were more likely to have STH infection, compared with students with good STH knowledge. There was no significant association of STH infection with sex, main source of drinking water, type of toilet, STH attitude score or practice (Table 4).

**Table 4:** Univariate and multivariate analysis assessing risk factors associated with any STH infection among schoolchildren in Laguna Province, the Philippines.

Characteristic	Total (N)	STH prevalence n (%)	Univariate		Multivariate	
			OR (95% CI)	*P-value*	AOR (95% CI)	*P-value*
**Age** ^a^	–	–	0.7 (0.7–0.8)	<0.001	1.5 (1.3–1.8)	<0.001
**Sex**						
Male	776	168 (21.6	1.0	–	–	–
Female	913	221 (24.2)	1.1 (0.9–1.4)	0.368	–	–
**SES** ^b^	–	–	1.2 (1.1–1.4)	<0.001	1.2 (1.1–1.3)	0.001
**Main source of water for drinking** ^c^						
Improved (piped water, bottled or mineral, hand pump)	1309	289 (22.1)	1.0	–	–	–
Unimproved (unprotected well, spring, river/pond)	291	77 (26.5)	1.2 (0.7–1.9)	0.598	–	–
No answer	89	23 (25.8)	–	–	–	–
**Type of toilet**						
Flush	392	78 (19.9)	1.0	–	–	–
Pour-flush or water-sealed toilet	1215	293 (24.1)	1.7 (0.7–3.9)	0.230	–	–
Closed Pit toilet (Antipolo)	40	11 (27.5)	1.7 (0.7–4.6)	0.267	–	–
No answer	42	7 (16.7)	–	–	–	–
**Rural/Urban**						
Urban	1237	322 (26.0)	1.0	–	1.0	–
Rural	452	67 (14.8)	0.5 (0.3–0.9)	0.019	0.7 (0.5–0.9)	0.016
**Overall knowledge score**						
Good	191	25 (13.1)	1.0	–	1.0	–
Poor	510	129 (25.3)	2.1 (1.3–3.5)	0.005	1.2 (1.0–1.6)	0.185
Average	988	235 (23.8)	1.9 (1.3–2.9)	0.002	1.2 (1.0–1.5)	0.013
**Overall Attitude Score**						
Good	377	91 (24.1)	1.0	–	–	–
Poor	531	123 (23.2)	1.0 (0.7–1.5)	0.992	–	–
Average	781	175 (22.4)	1.0 (0.7–1.3)	0.822	–	–
**Overall Practice Score**						
Good	1538	352 (22.9)	1.0	–		
Poor	13	5 (38.5)	2.8 (0.9–8.9)	0.089	–	–
Average	137	32 (23.4)	1.(0.8–1.7)	0.296	–	–

OR: Odds Ratio; AOR: Adjusted OR; CI: Confidence Interval.

^a^ Age as continuous variable in years (per 1 year increment).

^b^ SES: Socio-economic status. Factor score calculated using principal component analysis as described in Methods’ section. N = 1,425 with SES variable calculated that were included in the model.

^c^ The main water source was further re-categorised as improved/unimproved based on WHO/ UNICEF Joint Monitoring Programme (JMP) for Water Supply and Sanitation definition [40].

### Association between STH infection and nutritional indicators

Multivariate GEE regression modelling was used separately to assess the relationships between STH infection status and stunting; being underweight; and wasting (Table 5). Stunting (AOR: 1.3; 95% CI: 1.1–1.6; *P* = <0.001) and being underweight (AOR: 1.7; 95% CI: 1.2–2.3; *P* = 0.003), but not wasting (AOR: 1.2; 95% CI: 0.8–1.7; *P* = 0.375), were significantly associated with STH infection after adjusting for age, sex, SES, rural/urban classification of schools, presence of anaemia and within school clustering. Additionally, the multivariate GEE regression model was used to assess the relationship between STH and anaemia but no significant association (AOR: 1.1; 95% CI: 0.9–1.7; *P* = 0.462) was evident after controlling for age, sex, SES, rural/urban classification of schools and within school clustering (Table 6).

**Table 5:** Associations between any STH and nutritional indices among schoolchildren in Laguna Province, the Philippines based on a generalized estimating equations (GEE) logistic regression model.

Variable	Total (N)	Stunting n (%)	Univariate	Multivariate		Wasting	Univariate	Multivariate		Underweight ^c^		Univariate	Multivariate	
			OR (95% CI)	AOR (95% CI)	*P-value*	n (%)	OR (95% CI)	AOR (95% CI)	*P-value*	Total (N)	n (%)	OR (95% CI)	AOR (95% CI)	*P-value*
**Age** ^a^	–	–	1.7 (1.4–2.0**	1.5 (1.4–1.8)	<0.001	–	1.3 (1.1–1.6)**	1.5 (1.2–1.8)	0.001	–	–	1.3 (1.0–1.7)*	1.3 (1.0–1.7)	0.078
**Sex**														
Male	776	169 (21.8)	1.0	1.0	–	81 (10.4)	1.0	1.0	–	682	137 (20.1)	1.0	1.0	–
Female	913	173 (18.9)	0.8 (0.6 –1.1)	0.9 (0.8–1.1)	0.278	79 (8.7)	0.8 (0.5–1.2)	0.9 (0.6–1.4)	0.671	838	152 (18.1)	0.9 (0.6–1.2)	0.8 (0.6–1.1)	0.259
**SES** ^b^	–	–	1.3 (1.1–1.4)**	1.2 (1.1–1.3)	0.002	–	1.1 (0.9–1.2)	1.0 (0.8–1.1)	0.900	–	–	0.8 (0.7–0.9)**	1.2 (1.1–1.3)	<0.001
**Rural/Urban**														
Urban	1237	250 (20.0)	1.0	1.0	–	111 (9.0)	1.0	1.0	–	1,104	206 (18.7)	1.0	1.0	–
Rural	452	92 (20.4)	1.0 (0.8–1.3)	1.0 (0.9–1.2)	0.899	49 (10.8)	1.2 (0.8–1.8)	1.5 (0.9–2.2)	0.124	416	83 (20.0)	1.0 (0.7–1.2)	1.1 (0.9–1.6)	0.409
**Anaemia**														
Normal	1469	285 (17.6)		1.0	–	132 (9.0)	1.0	1.0	–	1324	236 (17.8)	1.0	1.0	–
Anaemic	220	57 (25.9)	1.5 (1.1–2.0)*	1.2 (1.0–1.4)	0.084	28 (12.7)	1.4 (1.0–2.1)	1.2 (0.8–1.8)	0.417	196	53 (27.0)	1.7 (1.1–2.5)*	1.7 (1.1–2.7)	0.021
**Any STH**														
Negative	1300	220 (16.9)	1.0	1.0	–	117 (9.0)	1.0	1.0	–	1204	203 (16.9)	1.0	1.0	–
Positive	389	122 (31.4)	2.3 (1.8–2.9)**	1.3 (1.1–1.6)	<0.001	43 (11.1)	1.2 (0.9–1.8)	1.2 (0.8–1.7)	0.375	316	86 (27.2)	1.8 (1.6–2.3)**	1.7 (1.1–2.3)	0.003

^a^ Age as continuous variable in years (per 1 year increment).

^b^ SES: Socio-economic status. Factor score calculated using principal component analysis as described in ‘Methods’ section; N = 1,425 with SES variable calculated that were included in the analysis

^c^ WAZ (as indicator of underweight) only calculated for up to 10 years of age (age group 10 years covers up to age 120 completed months because of its inability to differentiate between relative height and body mass beyond this age); N = 1,520

*P < 0.05

** *P*< 0.001 in univariable analysis

**Table 6:** Associations between any STH and anaemia among schoolchildren in Laguna Province, the Philippines based on a generalized estimating equations (GEE) logistic regression model.

Variable	Total (N)	Anaemia (N) (%)	Univariate		Multivariate	
			OR (95% CI)	*P-value*	AOR (95% CI)	*P-value*
**Age** ^a^	–	–	0.9 (0.8–1.1)	0.645	1.1 (0.8–1.2)	0.776
**Sex**						
Male	776	110 (14.2)	1.0	–	1.0	–
Female	913	110 (12.0)	0.9 (0.6–1.3)	0.454	0.9 (0.6–1.3)	0.722
**SES** ^b^	**-**	–	1.0 (0.9–1.1)	0.695	1.0 (0.8–1.1)	0.591
**Rural/Urban**						
Urban	1237	150 (12.1)	1.0	–	1.0	–
Rural	452	70 (15.5)	1.3 (0.8–2.2)	0.204	1.2 (0.8–2.0)	0.398
**Any STH**						
Negative	1300	162 (12.5)	1.0	–	1.0	–
Positive	389	58 (14.9)	1.2 (0.9–1.6)	0.305	1.1 (0.9–1.7)	0.462

^a^ Age as continuous variable in years (per 1 year increment).

^b^ SES: Socio-economic status. Factor score calculated using principal component analysis as described in ‘Methods’ section; N = 1,425 with SES variable calculated that were included in the analysis.

## Discussion

The results of this study showed that the overall prevalence of STH infection (23%) in the Laguna province study population remains above the WHO target of <20% for morbidity control. The true prevalence is likely to be higher due to the use of KK, which is known to decrease in sensitivity as prevalence and infection intensity decrease [11]. Nevertheless, the current prevalence estimate indicates that STH continues to be a public health problem among schoolchildren in this area. The most prevalent species present was *A*. *lumbricoides* (15.9%) followed by *T*. *trichuris* (13.3%), while a very small percentage of hookworm infections (0.12%) was recorded; this was not surprising given the low hookworm prevalence (6.8%) in Laguna province previously reported [11]. The majority of the schoolchildren had light intensity *A*. *lumbricoides*, *T*. *trichiura* infections or hookworm. The low infection intensity is likely attributable to the routine semi-annual deworming programme implemented in the study area.

Our KAP survey revealed that 64% of the schoolchildren claimed to be aware of intestinal worms, but follow-up questions demonstrated low levels of knowledge in some aspects. Misconceptions related to STH transmission, signs and symptoms, preventive measures and treatment among study participants were evident, indicating a lack of health education on STH. The majority of schoolchildren demonstrated an average attitude towards STH infection. This suggests the need for appropriate preventive programs to increase not only the knowledge of schoolchildren but also to improve their attitude towards STH. Although the majority of the study participants were categorized as having good practice regarding STH prevention, it was notable that a small proportion indicated they always defecated in the field or in a bush (3%) and in the river or canal (2%), indicating another gap in sanitary/hygienic practices. Of those who reported practicing open defecation, the majority (96%) recorded having a latrine/toilet at home. However, as we did not collect information on the functionality of sanitary facilities at home, it may be possible that not all children were able or willing to use a latrine or toilet in the home or at school.

In terms of potential risk factors for STH infection, our study showed that the risk of STH infection increased with age, emphasizing the importance of preventive chemotherapy in children during their primary school years, given that the peak prevalence and intensity occur during this period [41]. SES score was also found to be strongly associated with STH infections. In general, low SES reflects poverty which limits an individual’s access to safe, clean and adequate water supply and improved sanitation, and this results in an increased likelihood of transmission and maintenance of intestinal worms.

The odds of being infected were also higher among children with poor and average knowledge of STH. This underscores the importance of providing health education on STH in schools. Schools provide an opportune setting for delivering health information so that the most susceptible age groups are reached. Health education focusing on STH delivered in schools may help strengthen students’ knowledge and awareness about these parasites in general [42], including the perceived benefits of deworming [43].

The prevalence of any STH (*A*. *lumbricoides* and/or *T*. *trichiura)* was significantly higher in children attending urban compared to rural schools. This observation supports previous reports showing *A*. *lumbricoides* and *T*. *trichiura* are more prevalent in urban areas whereas hookworm is more common in rural areas [44–47]. This higher prevalence is likely due to overcrowding, inadequate supply of clean water and poor sanitation. Thus, regular deworming and health education programmes targeting students and their parents across both rural and urban settings should be emphasized. Although, the common source of transmission for the three STH species is through contaminated soil, it can be noted that the mechanism through which the infective stages enter the human host varies. For *A*. *lumbricoides* and *T*. *trichiura*, the route of transmission is through feacal-oral, while infection with hookworm (*N*. *americanus* and *A*. *duodenale*) can be acquired through skin penetration of larvae from feacal contaminated soil. Risk factors for *A*. *lumbricoides* and *T*. *trichiura* include inadequate access to clean water supply and ingestion of contaminated food [48]. Hookworm was more associated with inadequate sanitation but, in the case of *A*. *duodenale*, infection can also be acquired orally through contaminated food and water [48].

Despite the majority of the study participants reported to have access to household toilets and improved source for drinking water, the prevalence of any STH infections was still high. As earlier stated, since the information on the functionality of toilets and level of sanitary condition at the household level were not collected in the present study, it may be possible that these unmeasured factors facilitate STH transmission. The low prevalence of hookworm infection observed in this study however, may suggests that there is a limited feacal contamination in the external environment leading to a decreased risk of infection for hookworm within the study area. We did not observe any association between STH infection and the following variables: sex, main source of drinking water, type of toilet used, and attitude and practices towards STH among schoolchildren, findings contrasting with previous reports [49,50]. Risk factors associated with STH infections could, however, vary from one location to another depending on the area’s topographical landscape, environmental sanitation, lifestyle and culture of the resident population.

The prevalence of stunting (20.2%) and being underweight (19%) was lower than reported in the 2018 National Nutrition Survey of the FNRI where 25% and 24.5% of the schoolchildren (aged 6–10 years) were underweight and stunted, respectively [17]. These estimates were also found to be lower in previous studies conducted locally [24,25,51] and in other countries [52,53]. The prevalence of wasting (9.5%) in this study, however, was slightly higher than the 2018 national estimate of 7.6% [17] but lower than reported elsewhere [24,51–53]. Although, the prevalence estimates for stunting, being underweight and wasting reported in this study may be lower than previous estimates, they are still regarded as public health problems according to WHO criteria [54]. In addition, the prevalence of anaemia among study participants (13%) was classified as a mild public health problem based on WHO criteria and did not differ markedly from that reported in the 2018 national survey for children aged 9–10 years (12.4%) but was lower than reported in an earlier local study [28] and in other countries [52,53]. These discrepancies maybe due to differences in the dietary intake of the children, socio-economic factors, and/or other risk factors for malnutrition in schoolchildren [52]. The magnitude of malnutrition reported in this study indicates the need for interventions to avert the risk of malnutrition in schoolchildren.

It is notable that we found strong evidence of associations between any STH and stunting and underweight outcomes even with low prevalence of moderate to high intensity, after controlling for the effects of important confounders including age, sex, SES, rural/urban classification of schools, and the presence of anaemia and taking into account within school clustering. These outcomes demonstrate that schoolchildren infected with any STH were more likely to develop stunting and become underweight, results consistent with findings from other epidemiological studies involving schoolchildren in Malaysia [52], Peru [55] and China [56]. As shown previously, STH infection is associated with low food intake, impaired absorption of nutrients and altered metabolism resulting in poor growth and nutritional loss [7,57]. Although, there is not much evidence to support the malabsorption of nutrients as a mechanism contributing to underweight in STH-infected individuals, some reports have shown that *A*. *lumbricoides* lives in the gut where it can interferes with the nutritional absorption and cause damage to intestines resulting in symptoms such as diarrhoea, vomiting and consequently weight loss [7,57–59].

We also found SES as significant independent predictor for both stunting and underweight. This is expected given the long synergy between poverty and malnutrition. Meanwhile, we observed that the risk of stunting and wasting increased with age. Anaemia additionally was identified as a significant independent predictor of being underweight. This maybe due to an independent relationship between anaemia and being underweight (as shown to be related to micronutrient deficiency including iron deficiency [60,61]). We observed no significant relationship between STH and wasting, an unsurprising finding given that wasting is a measure of current or acute malnutrition; studies in Timor Leste [62] and South Ethiopia [53] showed similar results. Furthermore, this absence of effect may have been due to the high prevalence of light intensity infections.

Furthermore, we found no significant relationship between STH infection and anaemia after controlling for age, sex, SES, and rural or urban classification of schools. This lack of effect may have been due to the low prevalence of high-intensity STH infections, the low prevalence of hookworm infection, or other unmeasured risk factors for anaemia inherent in the study population. Anaemia in children can be caused by other contributing factors such as deficiencies in micronutrients, including iron, vitamin A, Vitamin B12 or folate, malaria or schistosome infection, or genetic risk (e.g., sickle cell, α-thalassemia) [63]; although the study area is not endemic for malaria or schistosomiasis, exclusion of the other variables in the model may have influenced the results we report.

Another potential limitation of this study was the cross-sectional design we employed. As a result, we were unable to assess any causal relationship between STH and the observed risk factors, or between STH infection and the indices of malnutrition. The STH infection reported here may not necessarily be indicative of previous or chronic infection. In addition, while we have adjusted for potential confounders in the analysis, the risk for residual confounding may be possible based on the risk of exposure to STH infection and nutritional indices and/or anemia not included in the SES variable. Data on parents’ level of education and/or family composition (i.e., crowding index) that might have been important in constructing the SES variable were not collected in this study, a research gap that needs to be considered in future investigations. The use of the KK procedure for the diagnosis of STH is also a limitation. Although recent studies suggest that the method is reasonably accurate for *A*. *lumbricoides* and *T*. *trichiura*, it can result in low sensitivity to detect hookworm infection, particularly when faecal samples are not examined immediately [11,64,65]. Despite these limitations, the study has important strengths such as the robust statistical approach employed with adjustment for school clustering and the multiple covariates in the models.

The current WHO strategy for STH control in children is to continually treat pre-school-aged children (PSAC) and school-aged children (SAC) once or twice a year depending on prevalence [35]. Questions have been raised on the potential benefits of deworming based on nutritional indicators, the impact on haemoglobin levels, cognition, school performance and mortality following a recent Cochrane Database Systematic Review by Taylor-Robinson et al., which concludes that “there’s substantial evidence that deworming does not improve average height, haemoglobin, cognition, school performance, or mortality” [66]. However, the report’s conclusions have been disputed due to the methods and study selection criteria employed [67–69] and/or concerns regarding whether systematic review methodology should be applied to STH studies due to the heterogeneity of STH prevalence of different species in different settings [69]. An article by Campbell et al. in 2016 [69] highlights the importance of appropriately designed studies that are powered to detect direct morbidity effects due to STH to strengthen evidence for deworming. Although the present study used cross-sectional data, the strength of associations between STH infection and child health outcomes (i.e., stunting and being underweight) observed in this study is striking and highlights the importance of continuous investigations on the impact of STH on child morbidity. Given that stunting and wasting are both indicator of long term (chronic) undernutrition (the latter’s an indicator for acute also), our cross-sectional data, may better reflect the accumulated morbidity of STH infection as opposed to RCTs or longitudinal studies with short period of follow-ups. To shed more light on this issue, future research should centre on the health effects of STH infections and of repeated doses of deworming provided over several years to assess the cumulative benefits of deworming.

The strong associations we report also suggest the need to address both STH infections and malnutrition in schoolchildren. There are existing programs currently available in the Philippines targeting STH infections (i.e., a nationwide school-based MDA program [16]) and malnutrition (school-based feeding program (SBFP) for severely wasted children [70]). Despite years of implementation, as consistently reported in a number of studies conducted across the country, the STH infection prevalence has continued to be high in schoolchildren [8–13] and nutritional improvements among children have not been sustained [70], indicating some challenges in the two programs. Although, it is difficult to draw firm conclusion about the impact of the MDA on STH infections levels from this study, the prevalence of moderate to heavy intensity infections observed in this study were lower compared to earlier studies, likely reflecting ongoing MDA. Thus, to ensure sustainable control of STH infections, other interventions are required. Our findings support the need for a holistic STH and nutrition programme in the Philippines. Uninterrupted deworming, complemented by provision of appropriate health education targeting parents and schoolchildren, improvements in sanitation facilities, and sanitation and hygiene practices, and a sustainable nutrition program, can provide the effective STH control measures required to improve the health and well-being of Filipino schoolchildren.

## Supporting information

S1 Table Attitude and practices of schoolchildren towards STH infection, in Laguna Province, the Philippines. (DOCX)

S1 Dataset Raw dataset and codebook. (XLSX)
