# Supplementary material for: Soil-transmitted helminth infections and nutritional indices among Filipino schoolchildren
Source: PLoS Negl Trop Dis. 2021 Dec 22;15(12):e0010008. doi: 10.1371/journal.pntd.0010008 (PMC8694453; doi:10.1371/journal.pntd.0010008)
Supplement: S1 Table — (DOCX) [file pntd.0010008.s001.docx]

Supplementary Material

S1 Table. Attitude and practices of schoolchildren towards STH infection, in Laguna Province, the Philippines

| **Attitude characteristics** | **No.** | % |
| --- | --- | --- |
| **Believe to be likely to get STH infection** |  |  |
| No | 104 | 6.2 |
| Yes | 1452 | 86.0 |
| Don't know | 130 | 7.7 |
| Missing | 3 | 0.2 |
| **Chance of getting STH** |  |  |
| Low possibility | 315 | 18.7 |
| Medium | 622 | 36.8 |
| High possibility | 210 | 12.4 |
| Don't know | 487 | 28.8 |
| Missing | 45 | 2.7 |
| **Will be worried if get infected with STH** |  |  |
| No | 184 | 10.9 |
| Yes | 1296 | 76.7 |
| Don't know | 191 | 11.3 |
| Missing | 18 | 1.1 |
| **STH will prevent/stop them from going to school** | |  |
| No | 718 | 42.5 |
| Yes | 688 | 40.7 |
| Don't know | 261 | 15.5 |
| Missing | 22 | 1.3 |
| **Can wash hands at school after toilet use** |  |  |
| No | 112 | 6.6 |
| Yes | 1446 | 85.6 |
| Don't know | 113 | 6.7 |
| Missing | 18 | 1.1 |
| **Overall Attitude Score** |  |  |
| Poor | 531 | 31.4 |
| Average | 781 | 46.2 |
| Good | 377 | 22.3 |
| ***Behaviour characteristics*** |  |  |
| **Wear slippers outside the house** |  |  |
| Always | 1384 | 81.9 |
| Usually | 192 | 11.4 |
| Rarely | 63 | 3.7 |
| Never | 41 | 2.4 |
| Missing | 9 | 0.5 |
| **Wear slippers inside the house** |  |  |
| Always | 735 | 43.5 |
| Usually | 387 | 22.9 |
| Rarely | 273 | 16.2 |
| Never | 289 | 17.1 |
| Missing | 5 | 0.3 |
| **Wear shoes at school** |  |  |
| Always | 1348 | 79.8 |
| Usually | 203 | 12.0 |
| Rarely | 101 | 6.0 |
| Never | 31 | 1.8 |
| Missing | 6 | 0.4 |
| **Place where he/she always defecate** |  |  |
| Home latrine | 1454 | 86.1 |
| Public/shared latrine | 70 | 4.1 |
| Field | 46 | 2.7 |
| River/canal | 29 | 1.7 |
| Don't know | 31 | 1.8 |
| Missing | 59 | 3.5 |
| **Wash hands after toilet use** |  |  |
| Always | 1250 | 74.0 |
| Usually | 247 | 14.6 |
| Rarely | 110 | 6.5 |
| Never | 65 | 3.8 |
| Missing | 17 | 1.0 |
| **Wash hands with soap after toilet use** |  |  |
| Always | 1383 | 81.9 |
| Usually | 184 | 10.9 |
| Rarely | 65 | 3.8 |
| Never | 33 | 2.0 |
| Missing | 24 | 1.4 |
| **Wash hands before eating** |  |  |
| Always | 1483 | 87.8 |
| Usually | 134 | 7.9 |
| Rarely | 53 | 3.1 |
| Never | 17 | 1.0 |
| Missing | 2 | 0.1 |
| **Wash hands with soap before eating** |  |  |
| Always | 1355 | 80.2 |
| Usually | 199 | 11.8 |
| Rarely | 83 | 4.9 |
| Never | 42 | 2.5 |
| Missing | 10 | 0.6 |
| **Wash fruits before eating** |  |  |
| Always | 1492 | 88.3 |
| Usually | 114 | 6.7 |
| Rarely | 43 | 2.5 |
| Never | 31 | 1.8 |
| Missing | 9 | 0.5 |
| **Cover left over food** |  |  |
| Always | 1381 | 81.8 |
| Usually | 144 | 8.5 |
| Rarely | 63 | 3.7 |
| Never | 98 | 5.8 |
| Missing | 3 | 0.2 |
| **Overall Behaviour Score** |  |  |
| Poor | 13 | 0.8 |
| Average | 137 | 8.1 |
| Good | 1538 | 91.1 |
